# AIDS: Ushering in a new era of shared responsibility for global health

**DOI:** 10.1186/1744-8603-8-26

**Published:** 2012-07-19

**Authors:** Kent Buse, Greg Martin

**Affiliations:** 1 UNAIDS, 20, avenue Appia, 1211, Geneva 27, Switzerland; 2 UNITAID, 20, avenue Appia, 1211, Geneva 27, Switzerland

**Keywords:** HIV, AIDS, Funding, Shared responsibility, Development cooperation

## Abstract

For the first time since AIDS erupted as worldwide emergency, global leaders, the scientific community, activists and people living with HIV are venturing to speak about the end to the pandemic. Signs of hope abound: over 8 million people are receiving life-saving treatment, the number of new infections is on significant decline, the remarkable evidence of treatment’s impact on preventing new infections and the aspiration of zero new HIV infections among children is firmly within grasp. This progress, won by people living with HIV and countries with support from partners such as the US programme PEPFAR, the Clinton Health Access Initiative and untold more, embodies global solidarity to bring about an AIDS-free generation. Shared responsibility and global solidarity represents a normative ideal to which both individual stakeholders and the global community must subscribe and embrace if our collective vision of an AIDS-free world is to be realised. The idea of shared responsibility and global solidarity needs to goes further than raising and investing resources and extend to the level of control countries take of their AIDS response. This editorial explores five areas that require further attention.

## 

For the first time since AIDS erupted as worldwide emergency, global leaders, the scientific community, activists and people living with HIV are venturing to speak about the end to the pandemic. Signs of hope abound: over 8 million people are receiving life-saving treatment, the number of new infections is on significant decline, the remarkable evidence of treatment’s impact on preventing new infections and the aspiration of zero new HIV infections among children is firmly within grasp. This progress, won by people living with HIV and countries with support from partners such as the US programme PEPFAR, the Clinton Health Access Initiative and untold more, embodies global solidarity to bring about an AIDS-free generation.

Yet, despite efforts made by countries, donor agencies and countless other global health initiatives as well as a series of ambitious commitments made by the international community in the 2011 Political Declaration on HIV/AIDS to be met by 2015 [1] – including among others to: (1) reduce sexual transmission by 50%; (2) eliminate new infections among children and reduce AIDS-related maternal deaths; (3) reach 15 million people with life-saving antiretroviral treatment; (4) reduce TB deaths among people living with HIV by 50 %; and (4)mobilise resources to meet the estimated annual investment need of US$ 22–24 billion-much remains to be done. The number of new infections continues to outpace the number of people newly accessing treatment [2]. The availability of appropriate formulations of medicines to treat children living with HIV is sorely lacking. Added to this, international financing for HIV has not increased since 2008. In part, this reflects the emergence of a new set of global challenges and priorities and in part, concerns held by those in the global North and South about how best to finance AIDS on a more sustainable basis (many national responses, particularly in Africa, are overwhelmingly dependent on external resources). This paradox of increasing aspiration and stagnating external financing sets the backdrop for the XIX International AIDS Conference in Washington DC this month. The twenty-some thousand conference goers will deliberate on the theme of “turning the tide” when the future of the AIDS response remains uncertain in the post-2015 development framework.

The AIDS movement has been a driver of innovation and political mobilization and has changed the face of global health over the past decades [3]. Moving forward, the movement must continue to innovate and press for a more clearly defined and deeper commitment to shared responsibility and global solidarity among countries and development partners. Indeed shared but differentiated responsibility was called for in the 2011 Political Declaration as a means for achieving its ambitious targets.

To that end, UNAIDS has suggested that shared responsibility and global solidarity rest on three premises:

 · Countries demonstrate political leadership through a willingness and ability to articulate a national AIDS, health and development vision and pull partner efforts in alignment.

 · Development partners and African governments fill the HIV investment gap together, through traditional and innovative means, investing “fair share” based on ability and prior commitments.

 · Resources are reallocated according to countries’ needs and priorities – among countries, programmes and populations – for greatest results while ensuring that responses promote human rights as well as synergies with other health and development efforts.

The idea of shared responsibility and global solidarity thus goes further than raising and investing resources. It extends to the level of control countries exert over their AIDS response. The Africa Union has unveiled a roadmap outlining steps that need to be taken for shared responsibility for AIDS to serve as a pathfinder to improve the health of Africans more broadly [4].

Many partners will need to play their part in ensuring the success of the African Union’s roadmap. UNITAID’s funding of projects focusing on the market dynamics of ARVs and HIV diagnostics, for example, will enable other programme to access lower prices and better quality products. Initiatives such as the Medicines Patent Pool may play an important role in ensuring that voluntary licenses allow for domestic production of ARVs. The flexibilities inherent in the TRIPS agreement, such as issuing compulsory licenses, should continue to be exploited where appropriate. Technology transfer, including south-south initiatives, should be encouraged to enable local medicines production and increase the array of supply companies. Mechanisms that allow for efficient registration of new drugs while assuring quality should be aggressively pursued – through support for the development of an African Medicines Regulatory Agency. The World Health Organization should ensure that the WHO Medicines Prequalification programme prioritizes the most needed medicines and continues to engage with NEPAD and other partners in local regulatory capacity building in African countries. Civil society, including networks of people living with and affected by HIV, have a critical role to play in continually pressing for action and accountability for results.

The promise of shared responsibility and global solidarity depends on how its challenges are addressed and the risks inherent in the transition are managed. We see five areas that require specific attention:

 1. Generating political leadership and ownership of responses that are evidence-informed and rights-based;

 2. Diversifying funding sources, including: transitional mechanisms for countries as they move towards increased domestic financing; options for new middle-income countries that still lack the fiscal space to fill the gap left by decreasing international support; new modes of cooperation with more established middle-income countries; and exploration of innovative sources;

 3. Enhancing regional integration to accelerate access to quality-assured and affordable medicines by: improving cross country ARV demand consolidation, forecasting and pooled procurement and; enabling leading pharmaceutical firms to serve regional markets by reducing trade barriers and enhancing harmonisation of national regulatory processes;

 4. Maintaining and enhancing the focus on human rights, gender equality and access to services for vulnerable populations;

 5. Enhancing accountability to address the risks associated with moving from performance-based, commodity-driven donor support to increased budget support and domestic financing, including through more inclusive governance.

Shared responsibility and global solidarity represents a normative ideal to which both individual stakeholders and the global community must subscribe and embrace if our collective vision of an AIDS-free world is to be realised.
